# Identifying artificial selection signals in the chicken genome

**DOI:** 10.1371/journal.pone.0196215

**Published:** 2018-04-26

**Authors:** Yunlong Ma, Lantao Gu, Liubin Yang, Chenghao Sun, Shengsong Xie, Chengchi Fang, Yangzhang Gong, Shijun Li

**Affiliations:** 1 Key Laboratory of Agricultural Animal Genetics, Breeding, and Reproduction of the Ministry of Education & Key Laboratory of Swine Genetics and Breeding of the Ministry of Agriculture, Huazhong Agricultural University, Wuhan, P. R. China; 2 Huadu Yukou Poultry Industry Co. Ltd, Pinggu, Beijing, P. R. China; Wageningen UR Livestock Research, NETHERLANDS

## Abstract

Identifying the signals of artificial selection can contribute to further shaping economically important traits. Here, a chicken 600k SNP-array was employed to detect the signals of artificial selection using 331 individuals from 9 breeds, including Jingfen (JF), Jinghong (JH), Araucanas (AR), White Leghorn (WL), Pekin-Bantam (PB), Shamo (SH), Gallus-Gallus-Spadiceus (GA), Rheinlander (RH) and Vorwerkhuhn (VO). Per the population genetic structure, 9 breeds were combined into 5 breed-pools, and a ‘two-step’ strategy was used to reveal the signals of artificial selection. GA, which has little artificial selection, was defined as the reference population, and a total of 204, 155, 305 and 323 potential artificial selection signals were identified in AR_VO, PB, RH_WL and JH_JF, respectively. We also found signals derived from standing and de-novo genetic variations have contributed to adaptive evolution during artificial selection. Further enrichment analysis suggests that the genomic regions of artificial selection signals harbour genes, including *THSR*, *PTHLH* and *PMCH*, responsible for economic traits, such as fertility, growth and immunization. Overall, this study found a series of genes that contribute to the improvement of chicken breeds and revealed the genetic mechanisms of adaptive evolution, which can be used as fundamental information in future chicken functional genomics study.

## Introduction

Chickens, like all other domestic animals, have a long history of artificial selection that dates back for centuries [1, 2]. Although the genetic information responsible for many notable changes in simple Mendelian traits have been successfully mapped, genetic signals for most economic traits that are modified during artificial selection are still unclear [3]. If successful, the detection of artificial selection signals, which is a central focus in the study of population genetics, can contribute to mapping the causal mechanisms related to economic traits in the genome and provide a clear understanding of how diversity evolved in various chicken breeds.

During domestication and subsequent commercial breeding, a variety of detectable selection signals were maintained in the genome [1, 4, 5]. Among them, signals shaped by artificial selection are consistently associated with some important economic traits during the process of breed formation. Given that genomic regions have a long-term divergent (bi-directional) selection, the extreme genetic differentiation in the respective regions will be generated and the corresponding allele frequency spectra will depart from what is expected under neutral conditions [6]. This is analogous to the diversity among domesticated, local and wild populations. In the twentieth century, domestic chickens have been refined to improve specialized economic traits for applications in modern breeding technologies based on quantitative genetics [1, 7]. Then, more and more commercial chicken lines, including both layer and broiler lines, have been bred with similar selection directions, which may share similar artificial selection evidence in specific genomic regions [2]. This genetic homogeneity was particularly promising in lines bred for similar purposes and selection pressure. Hence, they are excellent models for characterizing the genomic response to artificial selection of multiple populations.

With the recent implementation of high-throughput genotyping techniques for many populations, identifying selection signals at the genome level has become possible. In addition, there have also been many studies on statistical tests used for detecting selection signals based on different models [8–10]. The general model in which de-novo mutations arise and were selected to fixation within a short time frame is well known as hard sweep [7, 11–13]. In fact, a large number of identified signals with major effects can be categorized into this type of selection signal [7]. In addition to hard sweep, another selection signal model that signals for standing variation or reoccurring novel mutations at a locus has gradually gathered interest. These selection signals, known as soft sweeps, contrast with hard sweeps [7, 11–13]. So far, publications on soft sweep appear to be rare, but soft sweeps are likely to be prevalent in domestic animals, because they have almost no time to develop de novo mutations that mid-small effects that occur during the short process of artificial selection. In general, commercial traits due to human-driven selection are quantitative and have a polygenic basis, and they also respond rapidly under strong selection pressure [7]. Therefore, there is a good reason to believe that both soft sweep and hard sweep modes play an important role in the commercial breeding of economic traits.

In this study, we identified artificial selection signals using genome wide data from multiple chicken populations. In addition, we also tried to distinguish the potential artificial selection signals that are based on hard sweep from those resulting from soft sweep, and we found that the number of soft sweeps is greater than that of hard sweeps in the pool of artificial selection signals.

## Materials and methods

### Animals, genotyping data and quality control

All research involving animals was conducted under protocols (No. 5 proclaim of the Standing Committee of Hubei People’s Congress) approved by the Standing Committee of Hubei People’s Congress and the ethics committee of Huazhong Agricultural University in P. R. China. In addition, all experiments were performed in accordance with approved relevant guidelines and regulations. Genotypes obtained with the Affymetrix chicken 600 K Axiom-SNP-array were from a total of 331 individual chickens from nine different breeds, which included 110 Jingfen (JF), 82 Jinghong (JH), 12 Araucanas (AR), 16 White Leghorn (WL), 40 Pekin-Bantam (PB), 19 Shamo (SH), 16 Gallus-Gallus-Spadiceus (GA), 20 Rhinelander (RH) and 16 Vorwerkhuhn (VO). The breeds used have a wide geographical distribution and almost every major chicken category was represented, including layer, local breed, and chickens from wild population (Table 1). As typical representatives of the commercial line in this study, Jingfen and Jinghong chickens were bred in China to further improve egg-laying traits and have similar foundation stocks.

**Table 1:** Samples, origin, specialization and some characteristics of nine chicken populations used in this study.

Breed (abbreviation)	No. of SNPs^1^	Sample Size	Origin of samples	Specialization	Dwarf/ Normal	The other Breed Characteristics
**Vorwerkhuhn (VO)**	392,280	16	Germany	Local breed	Normal	Distinctive black and gold plumage;
**Araucanas (AR)**	392,280	12	Germany	Local breed	Normal	Blue eggs;
**White Leghorn (WL)**	392,280	16	Germany	Layer line	Normal	White shell; High production;
**Pekin-Bantam (PB)**	392,280	40	Germany	Local breed	Dwarf	Bantamized Cochin breed;
**Rhinelander (RH)**	392,280	20	Germany	Local breed	Normal	Medium size breed; mainly an exhibition breed today but originally used for egg-laying.
**Shamo (SH)**	392,280	19	Germany	Fancy breed	Normal	Game bird, heavy and upright posture;
**Gallus Gallus Spadiceus (GA)**	392,280	16	Thailand	Wild population	Normal	A subspecies of red junglefowl.
**Jinghong (JH)**	366,571	82	China	Layer line	Normal	Brown shell; High production;
**Jingfen (JF)**	364,449	110	China	Layer line	Normal	Brown shell; High production;

^1^ A total of 286,564 high-quality SNPs are shared in 9 populations after quality control.

Quality control of the SNP data was determined by the following criteria: (i) individual call rate > 0.95; (ii) SNP call rate > 0.99; (iii) SNPs in Hardy–Weinberg equilibrium in each breed (p>10e–6); (iv) SNP minor allele frequency > 0.01; (v) only autosomal SNPs with known positions were used. After assessing the quality control, we imputed the missing genotypes and inferred haplotypes using BEAGLE [14]. The final dataset contained 286,564 common SNPs that were genotyped in 331 individuals from 9 chicken populations.

### Population structure analysis

We employed the program ADMIXTURE v1.23 [15], which uses a block relaxation algorithm, to investigate the population structure with default parameters. The number of populations considered was from 2 to 12. To infer the phylogenetic tree, PLINK v1.07 [16] was used to generate the IBS distance for all pairs of individuals and then a neighbour-joining tree was constructed with FigTree v1.4.2 (http://tree.bio.ed.ac.uk/software/**figtree**/) based on the distance matrix. Finally, principal component analysis (PCA) was performed using the flashpcaR package[17] based on the whole genome genotype data that remained after quality control was assessed. After filtering SNPs to have a Pearson product-moment correlation of allele frequency (r^2^) less than 0.8 in 50 SNP windows, we ran ADMIXTURE and flashpcaR on all 231,545 remaining SNPs again.

### Linkage disequilibrium analysis

We calculated the correlation coefficient (*r*^2^) for every pair of SNPs to measure the LD level in each population using PLINK v1.07 [16]. The parameters were set as follows:—r2—ld-window-kb 1000—ld-window 99999—ld-window-r2 0. To visualize the LD decay in this analysis, the *r*^2^ values for 1000-bp distance bins were averaged and the corresponding figure was drawn by R script.

### Identifying signals under artificial selection

In accordance with the population genetic structure, five breed pools (or breed) of genotype data were used to detect potential selection signals in this analysis: Jinghong and Jingfen (JH_JF), Pekin-Bantam (PB), Rheinlander and White Leghorn (RH_WL), Araucanas and Vorwerkhuhn (AR_VO), Gallus-Gallus-Spadiceus (GA). To identify the signals under artificial selection, GA was defined as the common reference population because it was associated with limited amounts of artificial selection for commercial traits.

Multiple elementary selection signal statistics were employed to search for the evidence of artificial selection in two steps (hereafter termed the ‘two-step’ strategy). The first step is positive selection detection, which was performed using the composite likelihood ratio (CLR) [18] and the integrated haplotype score (iHS) [19] test. Among them, the CLR method calculates the likelihood ratio of selection signals by comparing the spatial distribution of allele frequencies in an observed window to the frequency spectrum of the whole genome. In this analysis, SweepFinder [18] software was employed to calculate the CLR with a grid size of 10 kb. To explore ongoing selection signals, the iHS method was performed, which searches for haplotype structures that have ancestral and derived alleles. Single marker scores for unstandardized iHS were calculated using the rehh R package [20] and then the |iHS| scores averaged across a non-overlapping 10 kb window across the genome. In the second step, we identified the underlying artificial selection signals from the above detected positive selection signals by calculating the F_ST_ statistic for pairwise sites between the observed populations and the common reference population. The unbiased F_ST_ estimate proposed by Weir and Cockerham was used to measure the population differentiation, with values ranging from 0 (no differentiation) to 1 (complete differentiation) [21]. To produce comparable CLR and iHS test results, the 10 kb grid size was also used for determining F_ST_ statistics. Finally, the selection signals were detected by using the |iHS|, CLR and F_ST_ scores in the genome level. The outliers for all methods were defined as signals with statistics surpassing the significance threshold (P<0.05) determined using 1,000 permutation tests. Accordingly, the chromosomal regions within a 200 kb window surrounding the outliers were defined as potential selection regions (PSR). This window size in this analysis was determined by the linkage disequilibrium decay in real data (S1 Fig). In addition, we further calculated the ‘observed heterozygosity’ (Het), which should be decreased in regions that are affected by a selection signal. As a by-product, the major (minor) allele frequency was also calculated by PLINK v1.07 [16]. Note that the artificial selection signals in this study were defined in the genomic region in which both positive selection signals and the F_ST_ statistical value were greater than the cut-off value at the genome level.

### Haplotype analysis

To characterize the core haplotype of underlying selection signals, haplotype blocks in the detected artificial selection regions were inferred using Haploview v4.2 [22] software based on the solid spin algorithm. The parameters were set as follows: -blockoutput SPI -blockSpineDP 0.8. Core haplotypes were defined as haplotype blocks that have a remarkable signal, and a high frequency in an observed population suggests that the presence of a core haplotype in the potential selection region may be abnormal in neutral scenarios. In general, in sweep analysis, the remarkable signals should be significant and the frequency should be greater than 0.8, according to previous reports [4].

### Functional annotation and gene ontology (GO) term enrichment analysis

In this analysis, bioinformatics analyses were performed to explore the potential biological functions of genes located in putative artificial selection regions. This analysis involved all the selected genes in the 200 kb window surrounding the significant signals, which was determined by the linkage disequilibrium decay in chicken populations. Detailed biological functions were matched for all highlighted candidate genes using the NCBI database (https://www.ncbi.nlm.nih.gov/gene). The gene annotation files for chickens that were downloaded from the database from ensemble (http://www.ensembl.org/info/data/ftp/index.html) were used to identify the exact position of markers in selected regions and highlight those that fell into selected regions using the textual mining R script. Gene-based annotations of Single nucleotide polymorphisms (SNPs) located in selected regions were performed using ANNOVAR [23]. Using ENSEMBL genes, we investigated where the SNPs are located in the regions of gene components. In addition, genes located in putatively selected regions were identified using the BioMart program (http://www.biomart.org/, Kasprzyk, 2011), and then an enrichment analysis, which included the terms cellular component, molecular function, and biological process, was performed for the identified genes using DAVID 6.7 (http://david.abcc.ncifcrf.gov/).

## Results and discussion

### Phylogenetic and population structure

**Fig 1:**
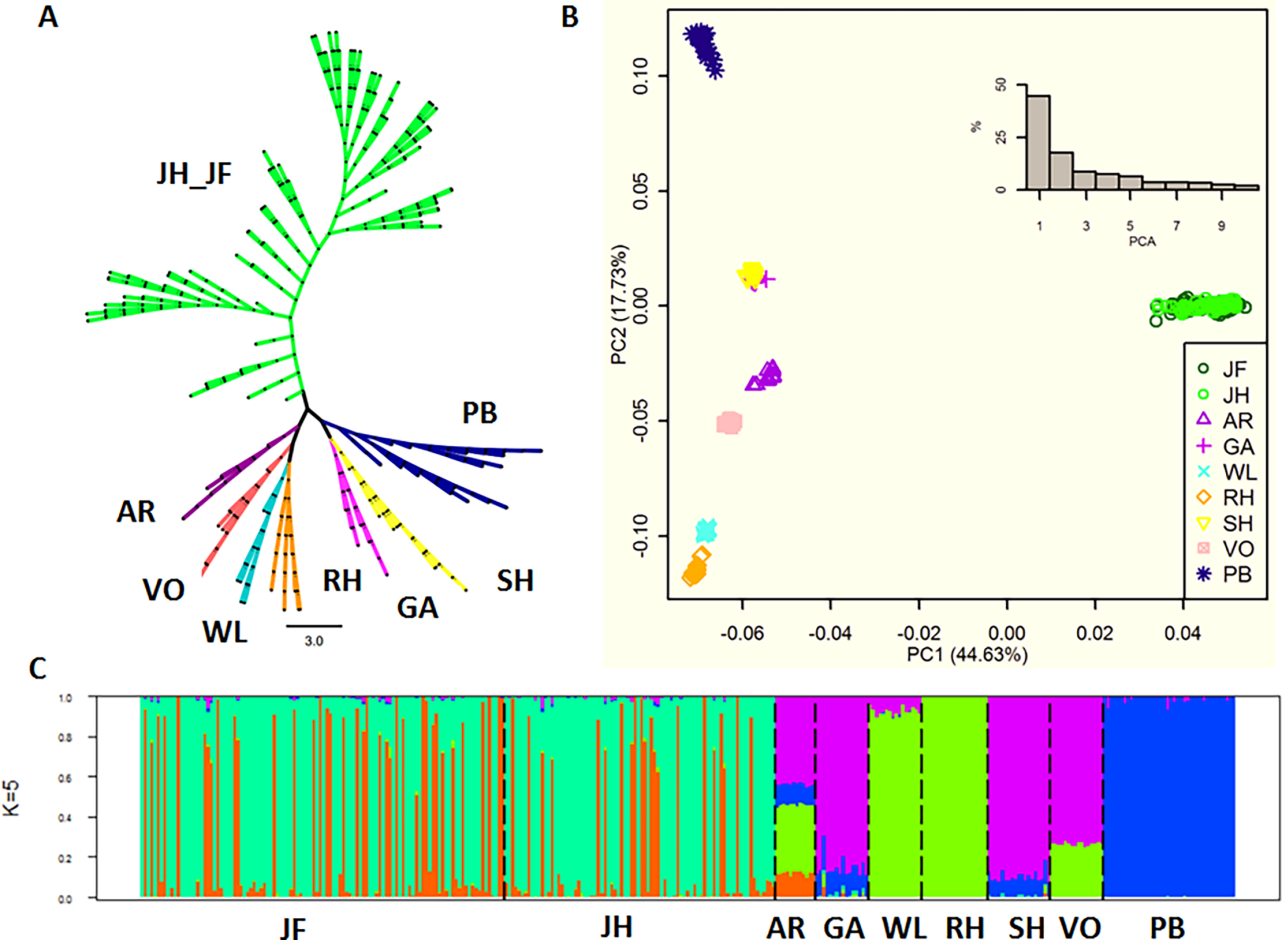
Analysis of the phylogenetic relationship and population structure of 9 chicken breeds. (A) Neighbor-joining tree constructed using SNP data. (B) Principal component analysis. (C) Population structure analysis of the 331 chicken individuals, showing the distribution of the K = 5 genetic clusters. See material and method for the abbreviations of the breeds.

After quality control assessment, a total of 286,564 high-quality SNPs were used to explore the genetic population structure and relatedness among 9 chicken breeds (Table 1). The neighbour-joining tree, based on IBD distance, showed that the Chinese commercial lines (JH_JF) were aggregated as a genetic group with a few mixed clades. In addition, the other 7 populations can be divided into 2 geographic groups as Asian breeds (PB, GA and SH) and Western breeds (AR, VO, WL and RH) (Fig 1A). These findings indicated that two Chinese commercial lines may be derived from the same ancestral population and that the geographic locations contribute to the genetic clusters.

In principal components analysis, two Chinese commercial lines formed a tight cluster, which further confirmed the findings from phylogenetic analysis. Western breeds and Asian breeds could be further subdivided into AR_VO (AR and VO) and RH_WL (RH and WL), and PB and GA_SH (GA and SH), respectively (Fig 1B). Among them, three layer groups, including JH_JF, PB and RH_WL, formed a congruent equilateral triangle around a centre wide group (GA_SH) in this analysis. Similar results were obtained when the markers that had a high degree of linkage disequilibrium after filtering the SNPs were used (S2 Fig). It may suggest that diversity is caused by a special geographic environment, commercial purpose or other unknown elements, which formed layers from the wide population with similar distances (Fig 1B).

In addition, the ADMIXTURE program was employed to investigate population structure and introgression, with the number of given populations (*K*) varying from 2 to 12 using all 286,564 SNPs and the 231,545 SNPs that remained after filtering for a high degree of linkage disequilibrium (Fig 1C, S3 and S4 Figs). As *K* increased, individuals were classified into the expected subpopulations based on the geographic position or the introgression event. However, no matter how the number of given populations changed, the two commercial lines were indistinguishable, which is consistent with the analysis above. It indicated that Jinghong and Jingfen are two commercial chicken lines from the same ancestral population, rather than two chicken breeds with an independent breeding history. Instead, the other populations can be clearly divided, especially when *K* is large enough, which suggests that those seven breeds have a long, independent breeding history compared with the two Chinese commercial lines. Note that the population structure analysis using PCA and the neighbour-joining tree method presented a similar result when *K* = 5 (or 4). This phenomenon indicated that some populations in this study may share a similar genetic basis. Therefore, Jinghong and Jingfen, Rheinlander and White Leghorn, Araucanas and Vorwerkhuhn were merged as JH_JF, RH_WL and AR_VO, respectively. Gallus-Gallus-Spadiceus (GA) was treated as the common reference population for identifying the artificial selection signals. It is possible that this combination strategy causes some positive selection signals for each breed to be lost. However, the drift can also mimic selection signals, especially if the effective population size is/has been small. Therefore, in this study, the pool population strategy based on the above analysis can reduce the impact of random drift with a little cost.

### Genome-wide artificial selection signals

**Fig 2:**
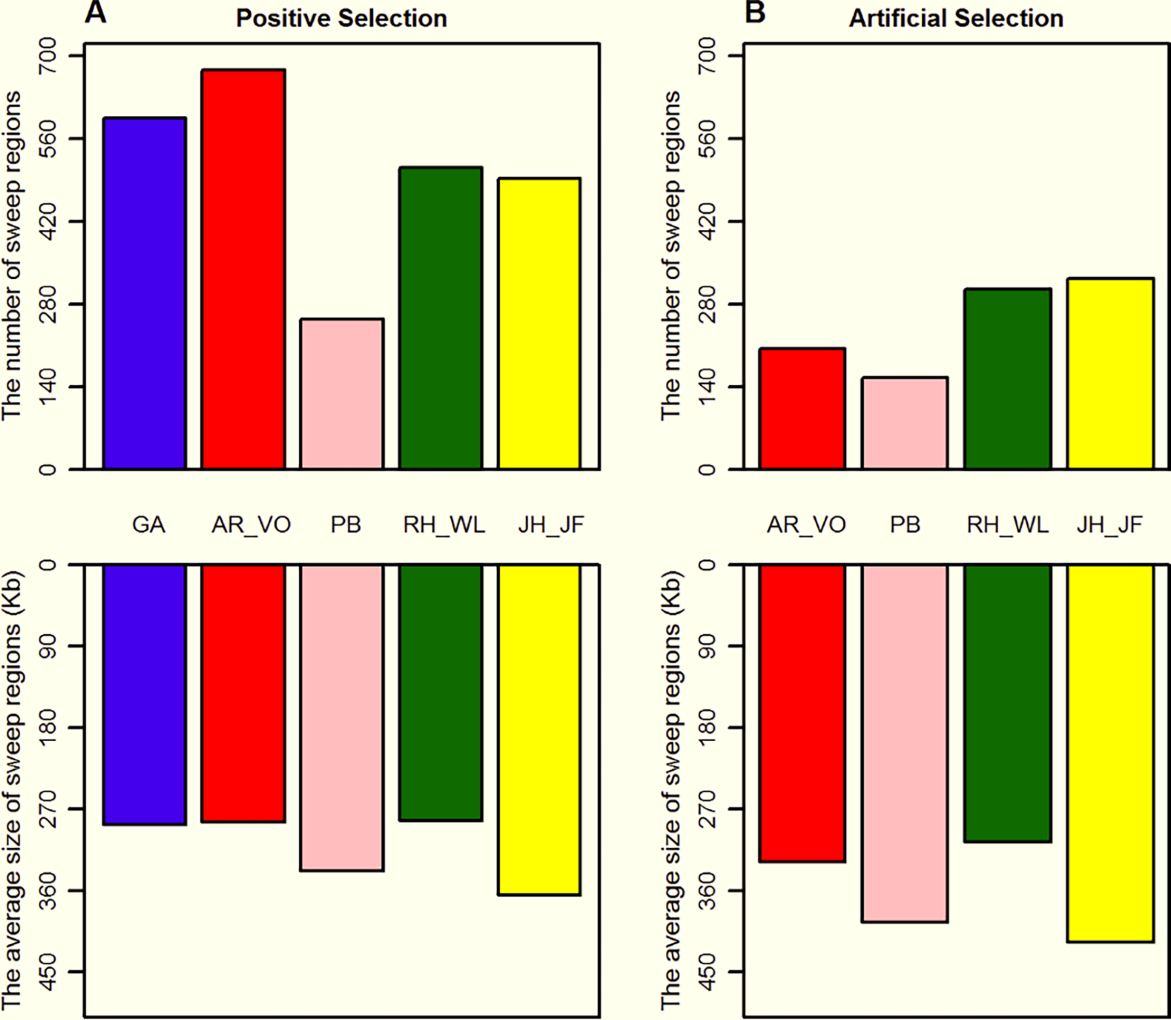
Visualization of the trend of sweep regions in all observed populations. (A) The potential positive selection regions identified by the iHS-CLR test. (B) The potential artificial selection regions identified by the ‘two-step method’, GA was defined as reference population in this analysis.

To detect positive selection, unstandardized iHS scores were calculated per site and the absolute values were averaged across non-overlapping sliding 10 kb windows across the whole genome. Correspondingly, the selection signals fell in 575, 626, 214, 365 and 194 potential selection regions in GA, AR_VO, PB, RH_WL and JH_JF, respectively. Similarly, a total of 26, 81, 79, 186 and 311 potential selection regions were also identified by the CLR test. In general, iHS is known to be good for detecting ongoing selection signals and CLR is most sensitive for detecting fixed selection signals. Therefore, the potential selection regions detected by iHS and CLR tests for each population were further merged when their interval distance was within a 200 kb window (hereafter termed iHS-CLR). Finally, a total of 595, 675, 254, 510 and 492 potential selection regions, spanning lengths of 170.93 Mb, 191.72 Mb, 85.90 Mb, 144.12 Mb and 179.26 Mb, were identified in GA, AR_VO, PB, RH_WL and JH_JF, respectively (Fig 2A).

**Fig 3:**
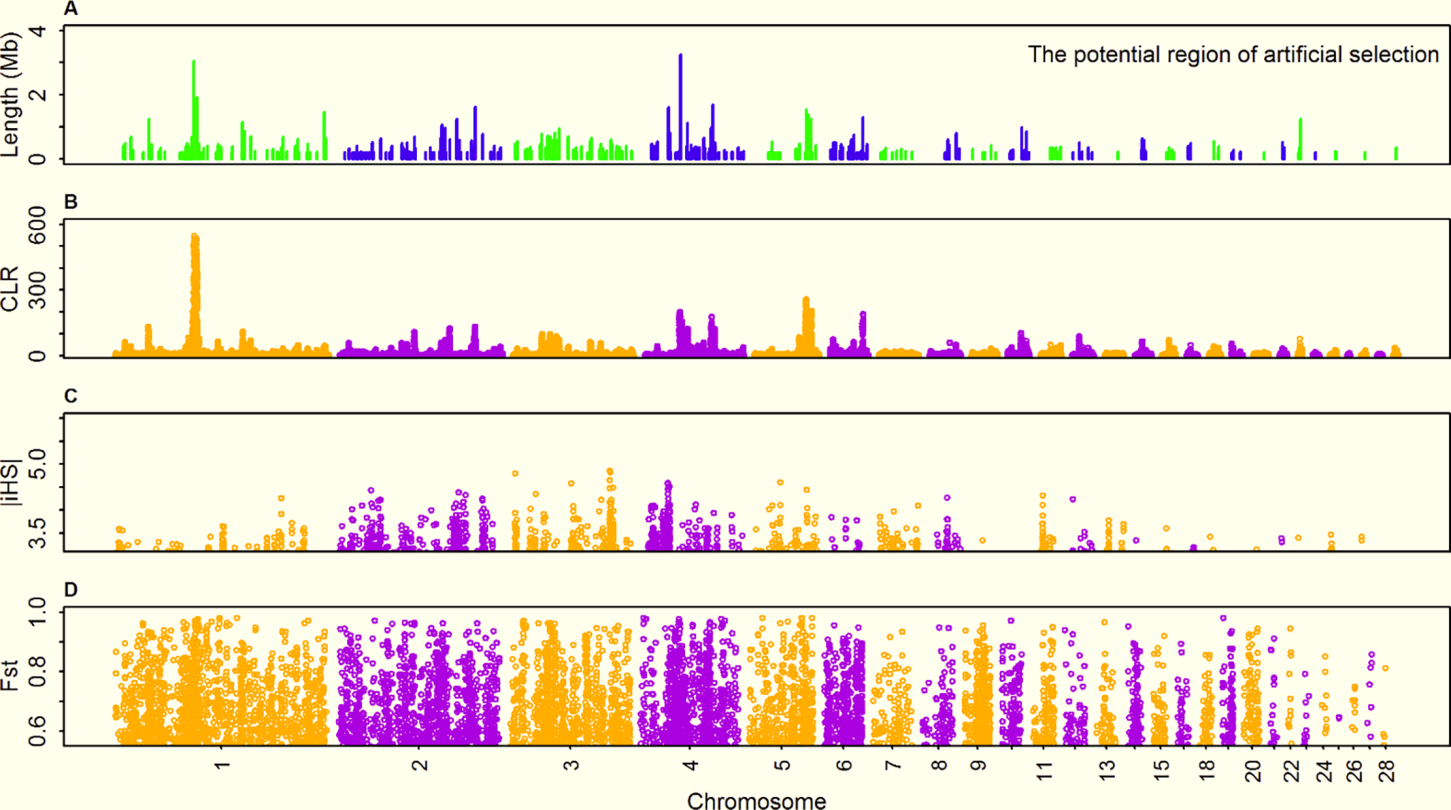
Summary of the genomic regions underlying artificial selection in JH_JF. (A) The lines illustrate the positions and lengths of genomic regions underlying artificial selection. (B, C, D) Manhattan plots based on CLR, iHS and F_ST_ tests, the y axis values are statistics scores, and the x axis shows positions along each chromosome.

To further identify the artificial selection signals, F_ST_ statistics were used to evaluate the differentiation between domestic and wild populations. When GA was treated as a common reference population, a total of 720, 1151, 1205 and 1057 selection regions containing the signals at the 5% significant level were found in AR_VO, PB, RH_WL and JH_JF, respectively. The overlapping selection regions detected by iHS-CLR and F_ST_ were defined as the artificial selection signals for further analysis. Collectively, 204, 155, 305 and 323 potential artificial selection signals spanning lengths of 66.90 Mb, 61.16 Mb, 93.28 Mb and 134.65 Mb were separately identified in AR_VO, PB, RH_WL and JH_JF compared with the wild population (Figs 2B and 3, S5 and S7 Figs). Remarkably, the average size of sweep regions underlying artificial selection is longer than all underlying positive selection (Fig 2). Although only about 54% (data ranged from 30.22% to 65.65%) of positive selection signals were shaped by artificial selection, the length of genomic fragments under artificial selection accounted for about 61.5% (ranged from 34.89% to 75.11%) of all positive selection regions. The results indicated that artificial selection is more powerful than natural selection in shaping the genome, resulting in rapid improvement of goal-directed economic traits in domestic populations[24].

To investigate the effects of selection on different gene components, we annotated where the selected SNPs are located in the genome. Out of 286,563 SNPs with an average density of 3.2 SNPs/kb on the genome, 37,737, 14,514, 15,425 and 21,671 SNPs fall into the artificial selection regions in JH_JF, PB, AR_VO and RH_WL, respectively. Taken JH_JF as an example, 31,520 were located in intergenic regions, 5,305 were in intronic regions and 350 were in exonic regions. Similar proportions of the SNPs that were located in different gene components were also observed in the genomic regions without artificial selection (S1 Table). Simultaneously, it seems that there is no difference among the effects of selection on regulatory region, coding sequence and the other gene components. We explain this phenomenon by the hitchhiking effect of selection that would shape the pattern of diversity around the selected locus and thus leaded to the difficulty of the causal selected locus mapping. However, the detailed annotation of SNPs in selected region is still helpful in facilitating chicken functional genomics study in the future (S2 Table).

Goal-directed economic traits in chicken were improved by human-driven selection in recent decades[1, 4]. Correspondingly, the genomic signals, such as long range haplotype homozygosity and skewed allele frequency spectra, would be generated under the great selection pressure[25]. From a statistical perspective, it’s difficult to distinguish the artificial selection signals from the positive selection signals only using a single method. By combining multiple methods, it is possible to identify artificial selection signals by utilizing both the wild and domestic populations. Our previous simulation study indicated that iHS and CLR statistics performed best and can be complementary in detecting positive selection signals [26]. The divergent signals that derived from wild population and further shaped by artificial selection can be detected by F_ST_ statistics. Accordingly, we used multiple methods to detect artificial selection signals to benefit from advantageous complementarities across methods in hope of improving the reliability of selection signals. In addition, the previous simulation studies also suggested that combining several methods can greatly increase the power to pinpoint the selected region [26, 27].

### Hard sweep and soft sweep

As described by Hermisson and Pennings [11], the signals underlying adaptive responses generally fall into two categories: hard sweep signals arise from newly arisen beneficial mutations and soft sweep signals arise from standing genetic variations. Chickens are an important animal economically, and most of their economic traits show quantitative heredity. In addition, the high polygenic basis for the important identified economic traits have been proven in recent genome-wide association studies [28, 29]. Therefore, we argue that selection of beneficial alleles already present in the population is the main reason for a rapid adaptation of economic traits in chickens.

To evaluate the contributions from standing genetic variations to artificial selection in chickens, we assumed the beneficial alleles for the important economic traits are still neutral or nearly neutral in Gallus-Gallus-Spadiceus. It may be closer to the ancestral population, but they have been prevalent in domestic populations because of artificial selection. Therefore, artificial selection regions with a haplotype allele frequency greater than 0.8 in domestic populations were reliable, and the corresponding signals based on emerging alleles (frequency is 0 in GA) and existing alleles (frequency is greater than 0.125, at least 4 gametes with the beneficial allele, in GA) were treated as a hard sweep and a soft sweep, respectively, in this study. In total, 10, 33, 43 and 113 potential hard sweeps of artificial selection (PAHSR) and 47, 60, 63 and 122 potential soft sweeps of artificial selection (PASSR) were identified in AR_VO, PB, RH_WL and JH_JF, respectively. It is generally known that artificial selection in chickens has yielded rapid changes in commercial traits that only have a history of approximately 100 years [1]. This means that commercial traits are likely to experience rapid improvement as a result of selection from standing genetic variation, especially when the trait is controlled by many mid-small effect loci [7]. However, there are also some mutations that have major effects that have been fixed or nearly fixed in domestic animals, such as mutations for double muscling in some beef cattle [30] and an *IGF2* mutation in pigs [31]. Therefore, the results may suggest that both standing and de-novo genetic variations have taken an active role in the process of artificial selection, probably at different time scales and with different selection coefficients. Here, we tried to disentangle hard sweep and soft sweep using wild populations with no strong artificial selection as a reference. However, there are several subspecies of red jungle fowl that have contributed to domestic chickens [32]. Therefore, the detected sweeps based on standing and de-novo genetic variations here are just one small part of them.

### A particularly interesting candidate selection region

**Fig 4:**
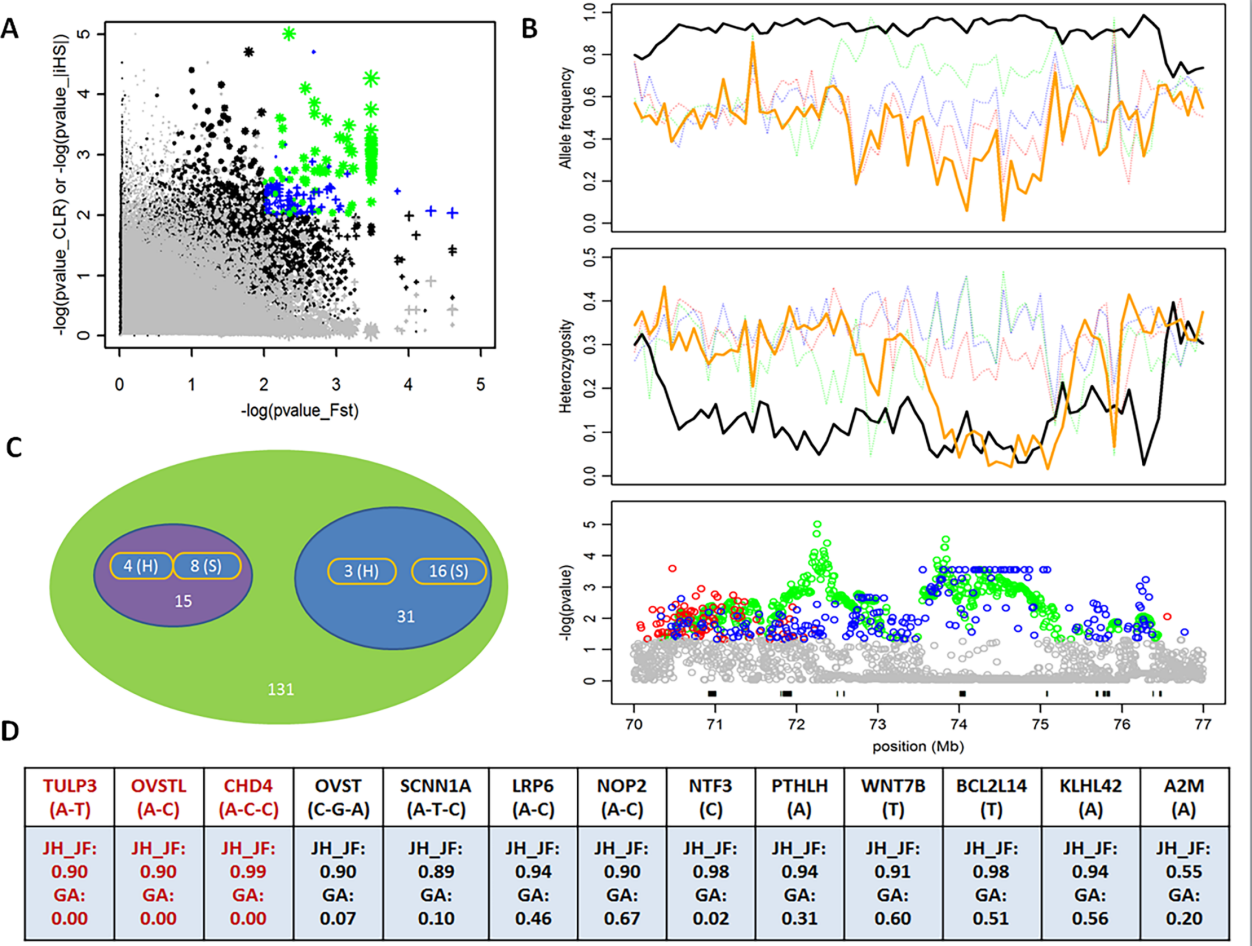
Highlight of a candidate selection region on GGA 1 in JH_JF. (A) Distribution of log10 (*P*_CLR_ or *P*_|iHS|_) and log10 (*P*_FST_) values calculated in sliding windows. The dots of color (corresponding to signals surpassing the 5% significance threshold of the empirical log10 (*P*_CLR_ or *P*_|iHS|_) and signals surpassing the 5% significance threshold of the empirical log10 (*P*_FST_) distribution) are genomic regions underlying artificial selection in JH_JF. The dots in green represent the significant signals located in this candidate selection region. (B) The bottom Manhattan plot shows all selection signals identified by CLR (the dots in green) and F_ST_ (the dots in blue) in the 70–77 Mb region of GGA1, respectively. The positions of 131 gene models in this region are displayed by the black segments. The middle plot shows the pattern of heterozygosity of four domestic populations in this region. The top plot shows the major allele frequencies in JH_JF and their corresponding frequencies in three other domestic populations in this region. The lines in black, orange, green, red and blue separately represents JH_JF, GA, PB, RH_WL and AR_VO. (C) Classification of 15 genes with multiple makers and 31 genes with single marker. H and S indicate hard sweep and soft sweep, respectively. (D) 13 genes and their core haplotype frequencies (or major allele) in JH_JF (Obs.) and GA (Ref.), See Supplementary S4 Table for the exact nucleotide positions.

We highlighted the genomic region in JH_JF with the strongest artificial selection signal (Fig 4A), which is located between positions 70–77 Mb on GGA1 and includes a total of 131 genes. As shown in Fig 4B, a series of remarkable positive selection signals in this region were identified by CLR analyses. The differentiation of loci between JH_JF and GA were detected by the F_ST_ test, which is suggestive of artificial selection during improvement of this commercial line. Notably, we also observed lower heterozygosity in this candidate selection region compared with background levels in GA from JH_JF. Correspondingly, the extreme differences in allele frequency between JH_JF and GA further show a strong indication of artificial selection in this region. The gradient variation of allele frequency in five populations indicated that divergent selection has further enriched the inter-population genetic diversity of chickens in this region. Out of 131 genes embedded in this candidate region, only 46 were overlapped with SNP markers in exon regions in this analysis. Among them, 31 genes were covered by a single SNP marker and the other 15 genes were covered by multiple SNP markers. In accordance with the above criteria, a total of 7 and 24 genes are believed to bind to hard sweep and soft sweep regions, respectively (Fig 4C). Finally, we further analysed the biological functions of the 46 genes located in this region using the available database. For JH_JF, at least 13 genes harboured in this region were related to egg quality [33–36], immune response [37], bone development [33], neuron functions [38, 39] and fertility [40] (Fig 4D). Notably, most of them correspond with the beneficial alleles in the reference population, with the exception of three genes that were selected from the novel present alleles when haplotype analysis was performed using the markers in the exon region. In conclusion, these results demonstrated that this genomic region was not only under strong artificial selection, but it also harboured soft and hard sweep, simultaneously.

### Underlying selected genes and Go terms for artificial selection

Based on the signals identified by the ‘two-step’ strategy, a total of 663, 668, 975 and 1394 genes were harboured in all artificial selection regions in AR_VO, PB, RH_WL and JH_JF, respectively. To further investigate the changes in biological process during artificial selection, those orthologous genes with human were used to perform an enrichment analysis using DAVID 6.7 (S3 Table). Most of terms did not give any intuitive information on artificial selection, such as some genes specific to PB and AR_VO were separate over-represented in the ‘ion transport’ (49 genes) and ‘low-density lipoprotein particle remodeling’(4 genes). However, Genes identified in JH_JF were enriched for the reproductive process, which mainly included ‘hsa04914: Progesterone-mediated oocyte maturation’ (14 genes), ‘hsa04114: Oocyte meiosis’ (14 genes) and ‘urogenital system development’ (15 genes), growth, which mainly included ‘regulation of growth’ (35 genes), and ‘growth’ (19 genes). These findings may reflect that artificial selection for JH_JF, a layer population, did improve the fertility and growth in recent decades. Similarly, genes found in RH_WL were mainly enriched for ‘embryonic development ending in birth or egg hatching’ (27 genes) and ‘in utero embryonic development’ (16 genes), which may contribute to fertility production. In addition, Gene Ontology (GO) analysis in four populations also revealed a series of biological process, including ‘behavioral fear response’ (3 genes in AR_VO), ‘behavior’ (42 genes in JH_JF), and ‘neuron development’ (32 genes in RH+WL), that are consistent with signal-related genes in this study. The findings may suggest that the signal-related genes and their corresponding biological processes may play an important role in changes of behaviour, habits, and commercial traits, such as modest temperament and the ability to grow fast.

Although there were no overall significant GO terms after Bonferroni correction, we found that genes related with a number of terms previously implicated in domestication-related changes are present within or close to these sweep regions. Among them, some candidate genes were observed that are related to immunity [37], neural system development [38], growth [41], feed intake [42] and fertility [40] (Table 2). We note that the established selective sweep around the *TSHR* gene in domestic chickens was also identified in our four populations. In general, this gene was considered as proof of principle demonstrating that the identification of selection signals should be reliable [1, 4]. Another interesting selection signal is located on GGA3 (105.92–106.09 Mb) and harbours the *NCOA1* gene, which is associated with total egg production [43]. For most layer history, egg production has consistently been treated as an objective trait of breeding. Correspondingly, the quality of the eggshell would also be considered and designed in the breeding programme. In this study, three putative genes (*PTHLH*, *OVST* and *SCNN1A*) overlapping with the strongest selection signals are related to eggshell characteristics. Among them, the *PTHLH* gene, located in the 72.51–72.52 Mb region of GGA1, plays an important role in calcium regulation[33]. As an estrogenic-stimulated gene involved in the process of oviduct development, the *OVST* gene is also associated with the formation of eggshells by regulating eggshell matrix protein secretion [35]. These results indicated that high levels of egg productivity would promote the evolution of calcium transportation system in layers. Note that the *SCNN1A* gene is precisely responsible for calcium transportation [34]. In contrast to egg production, another apparent characteristic of layer chickens is low weight. Here, a series of artificial selection signals at the *FGF6*, *TGFB2* and *IGF2* loci across different populations were detected. In accordance to previous reports [41, 44, 45], all of them are important candidate genes for growth traits. Remarkably, we also found three significant sweep regions that separately overlapped with the *PMCH* gene, which contributes to appetite [1], as well as the *KLHL42* and *LPCAT3* genes, which are related to feed intake [42, 46]. These findings indicated that the artificial selection of economic traits could have a cascading effect on phenotypes with genetic correlation, such as egg production, eggshell, growth, and feed intake. In addition, we also found a few genes with significant signals that are functionally plausible for neurodevelopment and immune response, which is consistent with previous studies of genes associated with chicken domestication [4].

**Table 2:** Some candidate genes overlap with the potential regions of artificial selection in four domestic chicken populations.

Chr.	Pos. (Mb)^1^	P-value. (method)^2^	Gene	Gene function
**1**	55.42–55.43	Fst0.039,*iHS0*.*025*;*Fst0*.*005*,*CLR0*.*002*; Fst0.008; *Fst0*.*035*;	*PMCH*	appetite, food intake[1]
**1**	70.84–70.94	*Fst0*.*027*,*iHS0*.*027*;*Fst0*.*005*;*Fst0*.*005*; Fst <0.001,Clr<0.001;	*WNT7B*	the developing central nervous system[38]
**1**	71.72–71.74	*Fst0*.*025*,*iHS0*.*017*;*Fst0*.*002*;*Fst0*.*005*,*iHS0*.*035*;Fst<0.001, Clr<0.001;	*BCL2L14*	immune[47]
**1**	72.42–72.44	*Fst0*.*025*,*iHS0*.*039*;*Fst<0*.*001*;*Fst0*.*005*,*iHS0*.*035*;Fst<0.001, Clr<0.001;	*KLHL42*	feed intake[42]
**1**	72.51–72.53	*Fst0*.*025*,*iHS0*.*039*;*Fst<0*.*001*;*Fst0*.*005*,*iHS0*.*035*;Fst<0.001, Clr<0.001;	*PTHLH*	bone development, eggshell[33]
**1**	73.16–73.17	*Fst0*.*026*,*iHS0*.*018*;*Fst<0*.*001*;*Fst0*.*001*;Fst<0.001,Clr<0.001;	*FGF6*	growth[41]
**1**	76.34–76.44	*Fst0*.*025*,*iHS0*.*004*;Fst0.001,*iHS0*.*006*; Fst0.031,*iHS0*.*004*,*CLR0*.*01*; Fst<0.001,Clr<0.001;	*OVST*	eggshell[35]
**1**	76.90–76.92	*Fst0*.*025*,*iHS0*.*003*;*Fst0*.*007*,*iHS0*.*008*;*Fst0*.*031*,*iHS0*.*004*,*CLR0*.*01*;*Fst0*.*013*,CLR<0.001;	*SCNN1A*	cation transportation, eggshell[34]
**1**	77.35–77.38	*Fst0*.*025*,*iHS0*.*003*;*iHS0*.*008*;*Fst0*.*015*,*iHS0*.*004*;*Fst<0*.*001*,*CLR<0*.*001*;	*LPCAT3*	feed intake[46]
**2**	14.04–14.88	*Fst0*.*042*,*iHS0*.*036*;Fst0.036,*CLR0*.*004*;*Fst0*.*007*,*iHS0*.*005*,*CLR0*.*014*; *Fst0*.*037*,iHS 0.041,*CLR0*.*014*;	*ITGB1*	skeletal myogenesis[48]
**3**	19.37–19.43	*Fst0*.*038*,*iHS0*.*048*; *Fst0*.*031*; *Fst0*.*004*;Fst 0.006, Clr 0.006;	*TGFB2*	growth traits[44]
**3**	105.92–106.1	Fst 0.019,*iHS0*.*013*; Fst 0.022; Fst 0.001; Fst 0.049;	*NCOA1*	total egg production[43]
**5**	13.77–13.78	*Fst0*.*009*,*iHS0*.*026*;*Fst0*.*031*;Fst0.001,*CLR0*.*017*;Fst0.004,*iHS0*.*008*;	*IGF2*	growth and carcass traits[45]
**5**	40.81–40.86	*Fst0*.*005*,*iHS0*.*004*,*CLR0*.*016*;*Fst<0*.*001*;*Fst<0*.*001*,CLR0.007; Fst <0.001, *CLR 0*.*004*;	*TSHR*	the reproductive machinery[1, 4]
**5**	49.02–49.03	*Fst0*.*006*,*iHS0*.*031*;*Fst0*.*005*;Fst0.036,iHS0.047;Fst<0.001,CLR<0.001;	*DLK1*	muscle hypertrophy[4]
**6**	14.82–14.84	*Fst0*.*032*,*iHS0*.*003;Fst0*.*001;Fst0*.*036*,*iHS0*.*023*,*CLR0*.*008;Fst0*.*013*,*iHS0*.*021*,*CLR0*.*008;*	*COMTD1*	Pigmentation[49]
**7**	22.54–22.55	*Fst0*.*038*,*iHS0*.*018;Fst0*.*022*,*CLR0*.*004;Fst0*.*004*,*iHS0*.*014;Fst0*.*033;*	*PRKAG3*	Meat quality[3]
**11**	16.18–16.63	*Fst0*.*037*,*iHS0*.*012;Fst0*.*005;Fst0*.*021;Fst<0*.*001*,*CLR0*.*007;*	*CDH13*	Disease-resistant[50]
**18**	4.47–4.51	*Fst0*.*006;Fst0*.*007;Fst0*.*004*,*CLR0*.*007;Fst0*.*004*,*CLR0*.*015;*	*PRPSAP1*	Abdominal fatness deposition[51]

^1^ This column presents the position of candidate genes which overlap with or close to the potential regions of artificial selection.

^2^ This column presents the genome-wide P-values of three statistics, corresponding to AR_VO, PB, RH_WL and JH_JF with semicolons.

## Conclusions

In this study, we identified the artificial selection signals based on nine chicken breeds using multiple statistical methods and further discussed the possible classifications of those signals. The successful application of the ‘two-step’ method suggested that artificial selection signals can be detected by combining multiple statistical methods and populations (or breeds). In some special cases, the pool population strategy based on the genetic relationship of multiple populations can reduce the impact from random drift in sweep analysis with the small cost of losing some signals for unique populations. According to the results, we found that both standing and de-novo genetic variations have contributed to adaptive evolution during the process of artificial selection. As a promising approach to studying population genomics, sweep analysis revealed a series of genes that contribute to the improvement of chicken breeds, which can be used as fundamental information in future chicken functional genomics study.

## Supporting information

S1 Fig Linkage disequilibrium (LD) decays for four domestic chicken populations. (TIFF)

S2 Fig Principal component analysis after filtering SNPs to have r2 greater than 0.8 in 50 SNP windows. (TIFF)

S3 Fig Population structure analysis of the 331 chicken individuals, showing the distribution of the K = 2–12 genetic clusters. (TIFF)

S4 Fig Population structure analysis of the 331 chicken individuals, showing the distribution of the K = 2–12 genetic clusters after filtering SNPs to have r^2^ greater than 0.8 in 50 SNP windows. (TIFF)

S5 Fig Summary of the genomic regions underlying artificial selection in AR_VO. (A) The lines illustrate the positions and lengths of genomic regions underlying artificial selection. (B, C, D) Manhattan plots based on CLR, iHS and F_ST_ tests, the y axis values are–log(P-value), and the x axis shows positions along each chromosome.
(TIFF)

S6 Fig Summary of the genomic regions underlying artificial selection in ZW. (A) The lines illustrate the positions and lengths of genomic regions underlying artificial selection. (B, C, D) Manhattan plots based on CLR, iHS and F_ST_ tests, the y axis values are–log(P-value), and the x axis shows positions along each chromosome.
(TIFF)

S7 Fig Summary of the genomic regions underlying artificial selection in RH_IT. (A) The lines illustrate the positions and lengths of genomic regions underlying artificial selection. (B, C, D) Manhattan plots based on CLR, iHS and F_ST_ tests, the y axis values are–log(P-value), and the x axis shows positions along each chromosome.
(TIFF)

S1 Table The summary of gene-based annotation. (DOC)

S2 Table The complete list of gene-based annotation of SNPs located in the potential selection regions. (XLSX)

S3 Table The complete list of four domestic populations’ enrichment analysis. (XLSX)

S4 Table The positions of SNPs overlap with the functional genes in the highlight candidate selection region on GGA 1 in JH_JF. (DOCX)
