# Supplementary figures and images for: Identifying artificial selection signals in the chicken genome
Source: PLoS One. 2018 Apr 26;13(4):e0196215. doi: 10.1371/journal.pone.0196215 (PMC5919632; doi:10.1371/journal.pone.0196215)

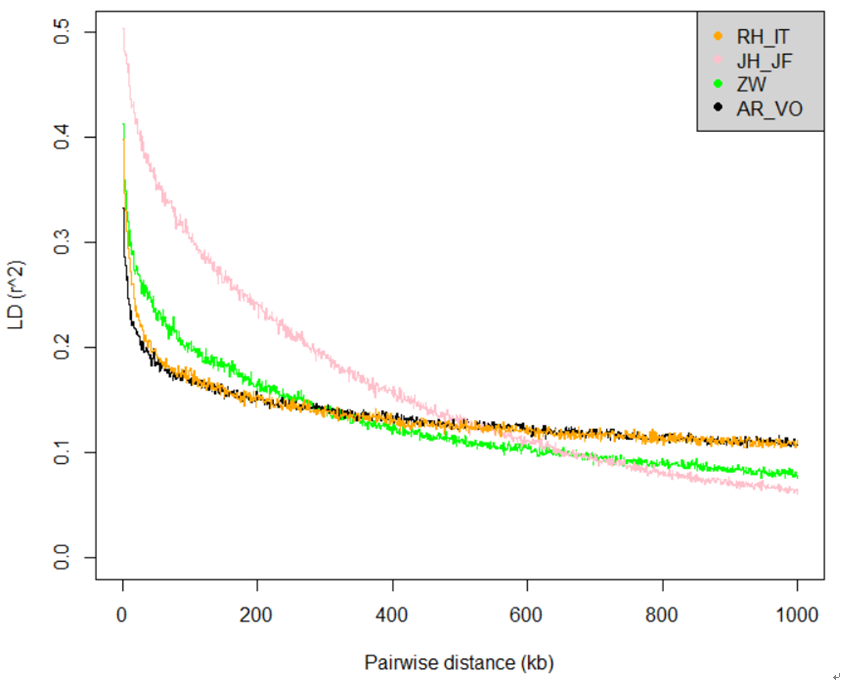

Supplement: S1 Fig — (TIFF) [file pone.0196215.s001.tiff]

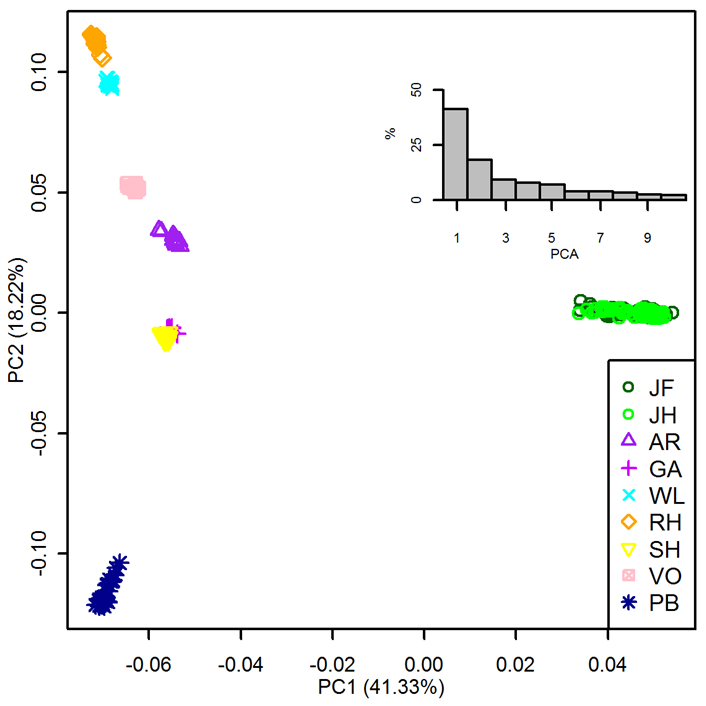

Supplement: S2 Fig — (TIFF) [file pone.0196215.s002.tiff]

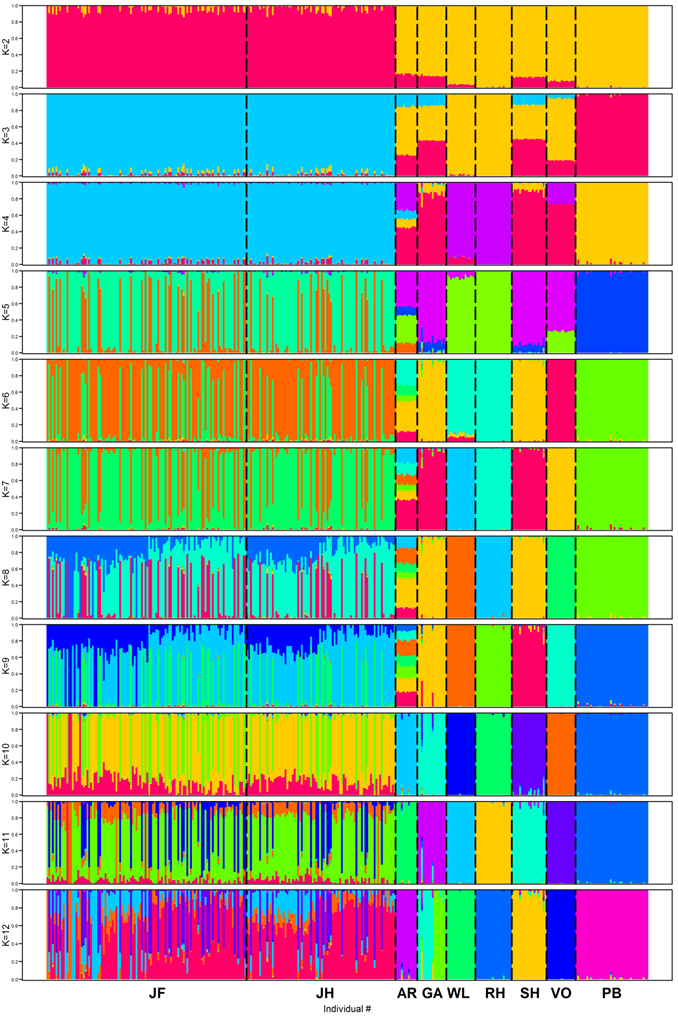

Supplement: S3 Fig — (TIFF) [file pone.0196215.s003.tiff]

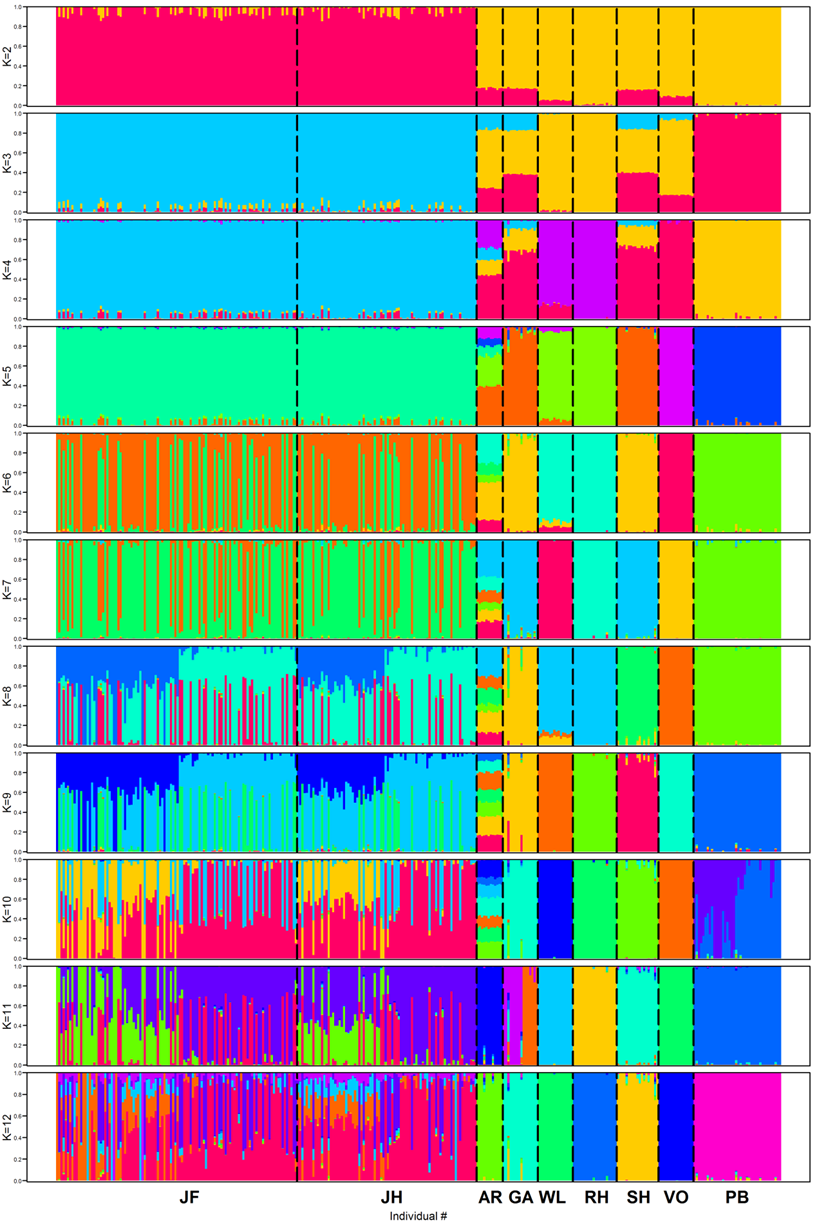

Supplement: S4 Fig — (TIFF) [file pone.0196215.s004.tiff]

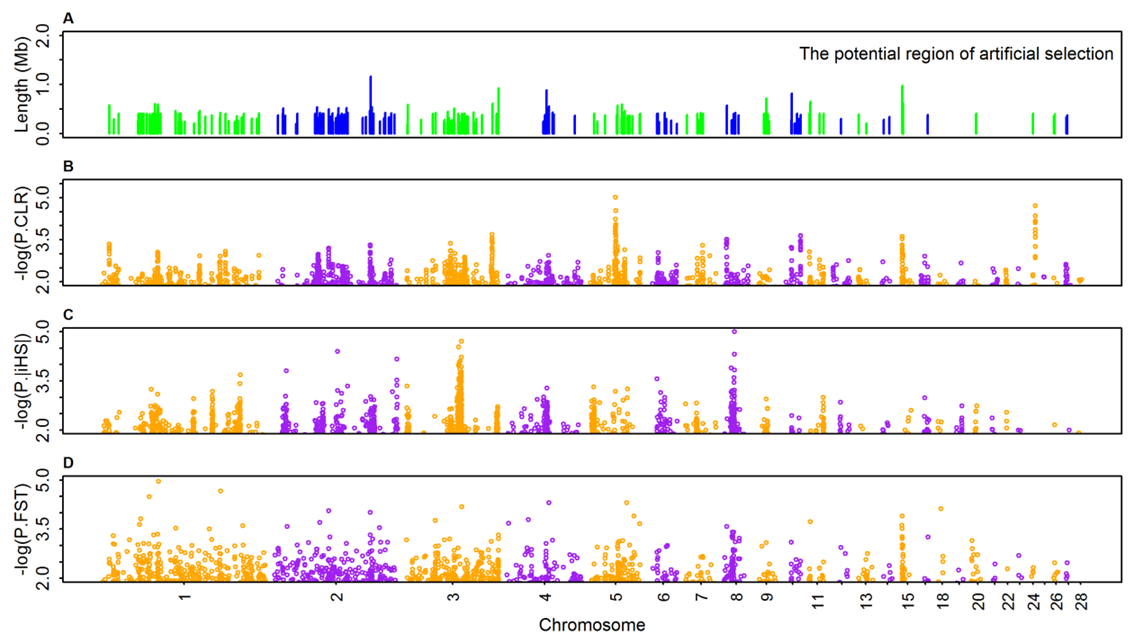

Supplement: S5 Fig — (A) The lines illustrate the positions and lengths of genomic regions underlying artificial selection. (B, C, D) Manhattan plots based on CLR, iHS and FST tests, the y axis values are–log(P-value), and the x axis shows positions along each chromosome. (TIFF) [file pone.0196215.s005.tiff]

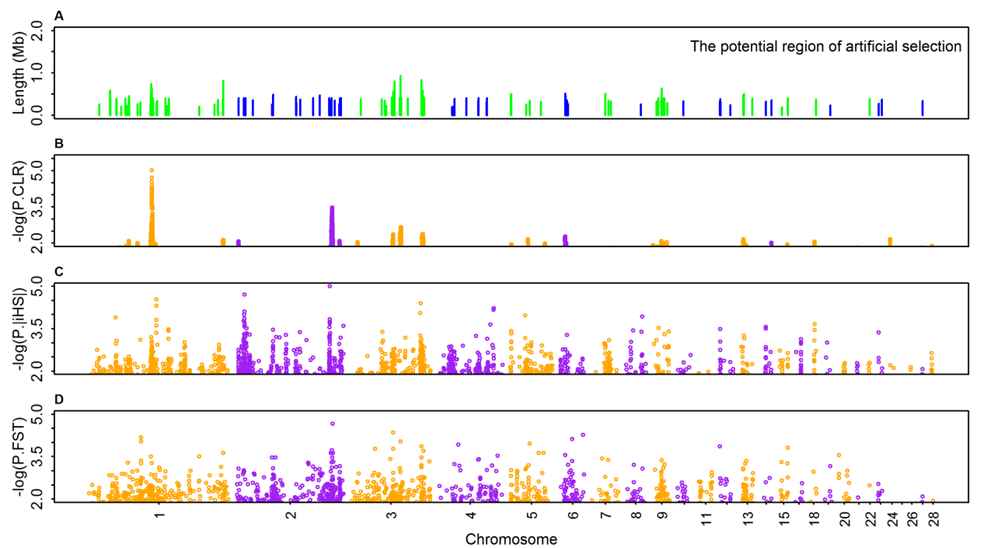

Supplement: S6 Fig — (A) The lines illustrate the positions and lengths of genomic regions underlying artificial selection. (B, C, D) Manhattan plots based on CLR, iHS and FST tests, the y axis values are–log(P-value), and the x axis shows positions along each chromosome. (TIFF) [file pone.0196215.s006.tiff]

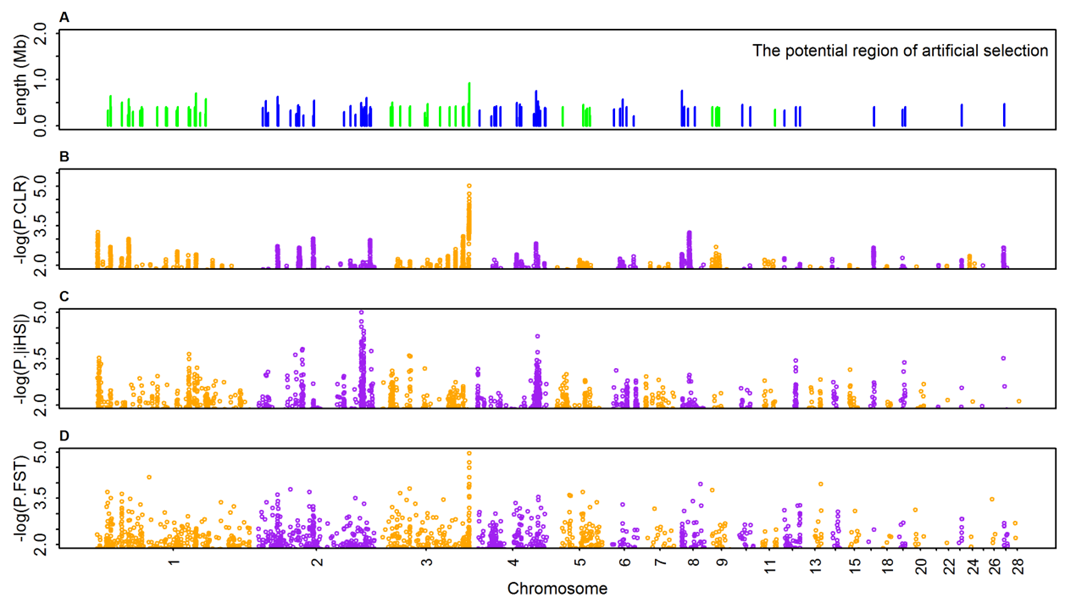

Supplement: S7 Fig — (A) The lines illustrate the positions and lengths of genomic regions underlying artificial selection. (B, C, D) Manhattan plots based on CLR, iHS and FST tests, the y axis values are–log(P-value), and the x axis shows positions along each chromosome. (TIFF) [file pone.0196215.s007.tiff]
