# Supplementary material for: Identifying artificial selection signals in the chicken genome
Source: PLoS One. 2018 Apr 26;13(4):e0196215. doi: 10.1371/journal.pone.0196215 (PMC5919632; doi:10.1371/journal.pone.0196215)
Supplement: S1 Table — (DOC) [file pone.0196215.s008.doc]

**S1 Table** The summary of gene-based annotation.

| **Category** | **ALL** | **NO SWEEP** | **JH_JF** | **PB** | **AR_VO** | **RH_WL** |
| --- | --- | --- | --- | --- | --- | --- |
| Intergenic | 232,679 | 161,414 | 31,520 | 14,514 | 15,425 | 21,671 |
| Intronic | 45,960 | 32,920 | 5,305 | 2,744 | 2,938 | 4,268 |
| ncRNA_intronic | 20 | 11 | 9 | 0 | 0 | 0 |
| Exonic | 2,938 | 2,168 | 350 | 181 | 155 | 213 |
| ncRNA_exonic | 25 | 20 | 4 | 0 | 0 | 1 |
| Splicing | 3 | 2 | 1 | 0 | 0 | 0 |
| UTR5 | 84 | 67 | 8 | 5 | 2 | 3 |
| UTR3 | 1,371 | 1,075 | 144 | 67 | 57 | 76 |
| Upstream | 1,517 | 1,165 | 171 | 88 | 62 | 80 |
| Downstream | 1,907 | 1,475 | 220 | 94 | 74 | 108 |
| Upstream/downstream | 60 | 51 | 5 | 2 | 2 | 1 |
| Total | 286,564 | 200,368 | 37,737 | 17,695 | 18,715 | 26,421 |
