# Supplementary material for: Identifying artificial selection signals in the chicken genome
Source: PLoS One. 2018 Apr 26;13(4):e0196215. doi: 10.1371/journal.pone.0196215 (PMC5919632; doi:10.1371/journal.pone.0196215)
Supplement: S4 Table — (DOCX) [file pone.0196215.s011.docx]

S4 Table. The positions of SNPs overlap with the functional genes in the highlight candidate selection region on GGA 1 in JH_JF.

| Gene | *TULP3*  (A-T) | *OVSTL*  (A-C) | *CHD4*  (A-C-C) | *OVST*  (C-G-A) | *SCNN1A*  (A-T-C) | *LRP6*  (A-C) | *NOP2*  (A-C) | *NTF3*  (C) | *PTHLH*  (A) | *WNT7B*  (T) | *BCL2L14*  (T) | *KLHL42*  (A) | *A2M*  (A) |
| --- | --- | --- | --- | --- | --- | --- | --- | --- | --- | --- | --- | --- | --- |
| Position | 75074458,  75084647 | 75822709,  75833498 | 76471406,  76482751, 76485341 | 75787259,  75791698,  75800843 | 76383957,  76389339,  76389797 | 71838527,  71839870 | 76464078, 7646514 | 74076957 | 72584139 | 70921255 | 71809825 | 72507879 | 76696294 |
| Frequency | JH_JF: 0.90  GA: 0.00 | JH_JF: 0.90  GA: 0.00 | JH_JF: 0.99  GA: 0.00 | JH_JF: 0.90  GA: 0.07 | JH_JF: 0.89  GA: 0.10 | JH_JF: 0.94  GA: 0.46 | JH_JF: 0.90  GA: 0.67 | JH_JF: 0.98  GA: 0.02 | JH_JF: 0.94  GA: 0.31 | JH_JF: 0.91  GA:0.60 | JH_JF: 0.98  GA: 0.51 | JH_JF: 0.94  GA: 0.56 | JH_JF: 0.55  GA: 0.20 |
